# A case report and literature review of Fanconi Anemia (FA) diagnosed by genetic testing

**DOI:** 10.1186/s13052-015-0142-6

**Published:** 2015-05-08

**Authors:** Ponnumony John Solomon, Priya Margaret, Ramya Rajendran, Revathy Ramalingam, Godfred A Menezes, Alph S Shirley, Seung Jun Lee, Moon-Woo Seong, Sung Sup Park, Dodam Seol, Soo Hyun Seo

**Affiliations:** Department of Paediatrics, Sree Balaji Medical College and Hospital, Chennai, 600 044 India; Department of Physiology/Central research laboratory (CRL), Sree Balaji Medical College and Hospital, Chennai, 600 044 India; College of Applied Medical Sciences and Molecular Diagnostics and Personalised Therapeutics Unit (MDPTU), Ha’il University, Ha’il, Kingdom of Saudi Arabia (KSA); Worked previously as in-charge and scientist in Central Research Laboratory (CRL), Sree Balaji Medical College and Hospital, Chennai, 600 044 India; Department of Laboratory Medicine, Seoul National University Hospital, Seoul, Korea

**Keywords:** Anemia, Fanconi Anemia, *FANC* genes, *FANCA* gene, Mutations

## Abstract

Fanconi anemia (FA) is a genetically heterogeneous rare autosomal recessive disorder characterized by congenital malformations, hematological problems and predisposition to malignancies. The genes that have been found to be mutated in FA patients are called *FANC*. To date 16 distinct *FANC* genes have been reported. Among these, mutations in *FANCA* are the most frequent among FA patients worldwide which account for 60- 65%. In this study, a nine years old male child was brought to our hospital one year ago for opinion and advice. He was the third child born to consanguineous parents. The mutation analyses were performed for proband, parents, elder sibling and the relatives [maternal aunt and maternal aunt’s son (cousin)]. Molecular genetic testing [targeted next-generation sequencing (MiSeq, Illumina method)] was performed by mutation analysis in 15 genes involved. Entire coding exons and their flanking regions of the genes were analysed. Sanger sequencing [(ABI 3730 analyzer by Applied Biosystems)] was performed using primers specific for 43 coding exons of the *FANCA* gene. A novel splice site mutation, c.3066 + 1G > T, (IVS31 + 1G > T), homozygote was detected by sequencing in the patient. The above sequence variant was identified in heterozygous state in his parents. Further, the above sequence variant was not identified in other family members (elder sibling, maternal aunt and cousin). It is concluded that genetic study should be done if possible in all the cases of suspected FA, including siblings, parents and close blood relatives. It will help us to plan appropriate treatment and also to select suitable donor for hematopoietic stem cell transplantation and to plan for genetic counseling. In addition to the case report, the main focus of this manuscript was to review literature on role of *FANCA* gene in FA since large number of *FANCA* mutations and polymorphisms have been identified.

## Background

Fanconi anemia (FA) is a genetically heterogeneous rare autosomal recessive disorder characterized by congenital malformations, hematological problems and predisposition to malignancies [1]. The genes that have been found to be mutated in FA patients are called *FANC*. To date 16 distinct *FANC* genes (*FANCA, FANCB, FANCC, FANCD1 (also known as BRCA2), FANCD2, FANCE, FANCF, FANCG, FANCI, FANCJ, FANCL, FANCM, FANCN, FANCO, FANCP, and FANCQ)* have been reported [2-5]. Among these, mutations in *FANCA* are the most frequent among FA patients worldwide which account for 60- 65% [2,6-8]. As large number of *FANCA* mutations and polymorphisms have been identified, the *FANCA* is considered as a hyper-mutable gene [2,6,7,9-12]. In this study targeted next-generation sequencing (MiSeq, Illumina method) was performed by mutation analysis in 15 genes- *BRCA2*, *BRIP1*, *FANCA*, *FANCB*, *FANCC*, *FANCD2*, *FANCE*, *FANCF*, *FANCG*, *FANCI*, *FANCL*, *FANCM*, *PALB2 (FANCN), RAD51C (FANCO), and SLX4 (FANCP)*, then novel non-synonymous variants were confirmed by Sanger sequencing (ABI 3730 analyzer by Applied Biosystems). Entire coding exons and their flanking regions of the genes were analysed. Sanger sequencing was performed using primers specific for 43 coding exons of the *FANCA* gene. A novel splice site mutation, c.3066 + 1G > T, (IVS31 + 1G > T), homozygote was detected by sequencing in the patient. The above sequence variant was identified in heterozygous state in his parents. Further, the above sequence variant was not identified in other family members (elder sibling, maternal aunt and cousin). In addition to the case report, the main focus of this manuscript was to review literature on role of *FANCA* gene in FA since large number of *FANCA* mutations and polymorphisms have been identified.

## Case presentation

### Clinical report

A nine years old male child was brought to us one year ago for opinion and advice. He was already seen in many other tertiary care centres and was diagnosed to have Fanconi Anemia (FA) at 8 years of age. The subject’s cytogenetic report was positive for mitomycin-C (MMC) induced chromosomal aberrations. Since then he has been getting blood transfusion, now almost once in a month. He is the third child born to consanguineous parents. The first child was a male and was diagnosed to have FA at 8 years of age. He was on anabolic steroids and periodic blood transfusions but died at 10 years of age due to intracranial bleed. The second child is also a male and is now 20 years old and normal.

The index case was born at term by Lower Segment Caesarean Section (LSCS) and was small for gestational age. The birth weight was 2.3 Kg. He used to get recurrent respiratory infections and feeding problems. The mother noticed that his heart was beating abnormally and hence an Echocardiogram (ECHO) was done at five months of age and was found to have Patent Ductus Arteriosus (PDA). It was ligated. After 1 year of age he had recurrent Urinary Tract Infection (UTI). Investigations revealed that he had chronic atrophic pyelonephritis of right kidney due to Vesico Ureteric Reflux. Micturating Cystourethrography (MCU) showed Grade IV reflux on right side and Grade III reflux on left side. Hence Sting procedure was done. Subsequent scan done showed that the left kidney was functioning normally. At 3 years of age he developed bruising on right thigh. Investigations done at 8 years of age showed that he had FA. Since then he has required platelets and packed red cells, now almost every month.

On examination, his weight was 30 Kg (Expected – 29 Kg), Head circumference – 51 cm (Expected – 53 cm), Height 133 cm (Expected – 131 cm), Upper segment was 59 cm; Lower segment was 74 cm. External genitalia was normal. Patient had pallor, left sided squint, high arched palate, short neck, mild gynecomastia, hyperpigmentation of nail beds and palms. The cardiovascular system (CVS) examination revealed grade 2/6 soft systolic murmur. Other systems were normal.

We were interested in performing genetic study of the case which would also help in proper genetic counselling to the family. After obtaining informed consent from the family 2 mL ethylene diamine tetra-acetic acid (EDTA) blood was collected from proband, parents, elder sibling and the relatives [maternal aunt and maternal aunt’s son (cousin)] for mutation analysis. The extracted DNA was sent to the Department of Laboratory Medicine, Seoul National University Hospital, Seoul, Korea for mutation analysis under collaboration study.

### DNA extraction for molecular genetic testing

#### DNA extraction

After obtaining informed consent, 2 mL ethylene diamine tetra-acetic acid (EDTA) blood samples were collected from proband, parents, elder sibling and the relatives [maternal aunt and maternal aunt’s son (cousin)]. The genomic DNA was isolated from white blood cells by Phenol: Chloroform: Isoamylalcohol method [13].

### Molecular genetic testing

The targeted next-generation sequencing (MiSeq, Illumina method) was performed by mutation analysis using a multigene panel consisted of 15 genes- *BRCA2*, *BRIP1*, *FANCA*, *FANCB*, *FANCC*, *FANCD2*, *FANCE*, *FANCF*, *FANCG*, *FANCI*, *FANCL*, *FANCM*, *PALB2 (FANCN), RAD51C (FANCO), and SLX4 (FANCP)* [14]. Entire coding exons and their flanking regions of the genes were analysed. The reference sequences have been mentioned in Table 1. Sequences were captured by TruSeq Custom Enrichment Kit (Illumina) and sequenced with MiSeq (Illumina). Among the variants detected, all previously reported mutations and probable pathogenic variants including novel non-synonymous ones were confirmed by Sanger sequencing, as well as all low-coverage regions with coverage depth under 10X. Sanger sequencing was performed using primers specific for 43 coding exons of the *FANCA* gene. The amplified products were sequenced on an ABI 3730 analyzer (Applied Biosystems, Foster City, CA, USA) using a BigDye Terminator v3.1 Cycle sequencing kit (Applied Biosystems). The variant were queried against the public version of the Human Gene Mutation Database (HGMD, http://www.hgmd.cf.ac.uk/). For nucleotide numbering Nucleotide c.1 was the “A” of the initiation codon and for Amino acid numbering p.1 was the initiation codon, ATG.

**Table 1:** **The reference sequences used for the 15 genes in molecular genetic testing of Fanconi Anemia**

**Gene**	**Reference sequence**	**Gene reference**	**Sequence**
BRCA2	NG_012772.3, NM_000059.3	FANCG	NG_007312.1, NM_004629.1
BRIP1	NG_007409.2, NM_032043.2	FANCI	NG_O11736.1, NM_001113378.1
FANCA	NG_011706.1, NM_000135.2	FANCL	NG_007418.1, NM_018062.3
FANCB	NG_007310.1, NM_001018113.1	FANCM	NG_007417.1, NM_020937.2
FANCC	NG_011707.1, NM_000136.2	PALB2	NG_007406.1, NM_024675.3
FANCD2	NG_007311.1. NM_033084.3	RAD51C	NG_023199.1, NM_058216.1
FANCF	NG_011708.1, NM_021922.2	SLX4	NG_028123.1, NM_032444.2
FANCF	NG_007425.1, NM_022725.3		

## Results

### Analysis of *FANCA* mutation

**Figure 1:**
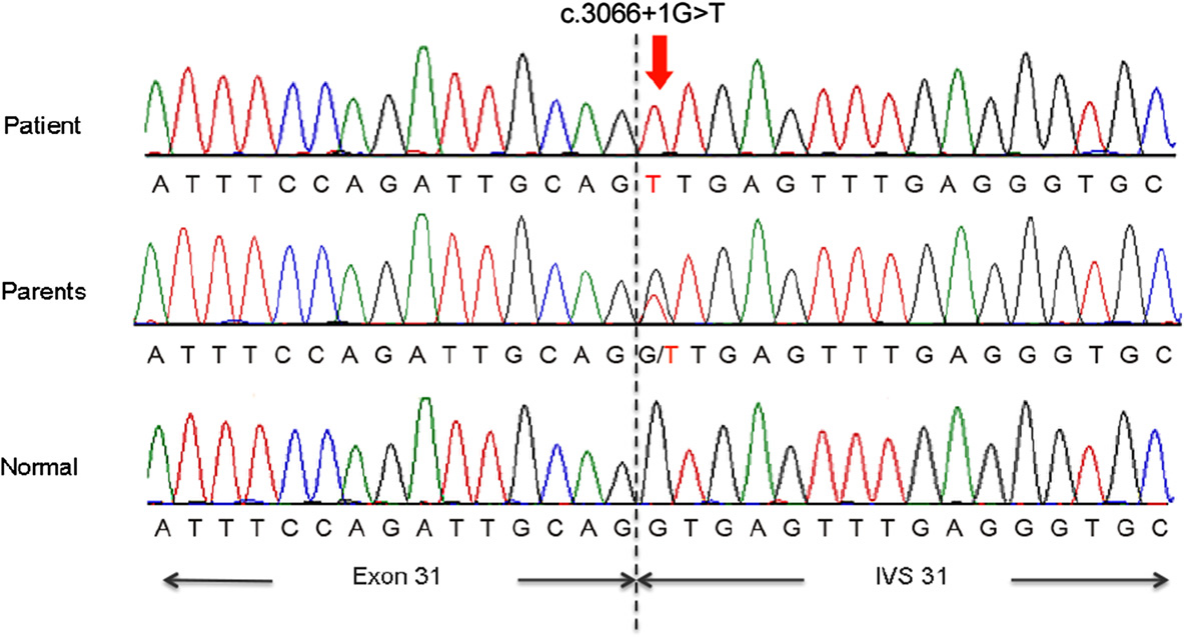
*FANCA* mutation- A novel splice site mutation, c.3066+1G>T, detected in the proband and heterozygous mutation identified in both parents of proband, shown as electropherograms (Reference cDNA sequence: NM_000135.2).

A novel splice site mutation, c.3066 + 1G > T, (IVS31 + 1G > T), homozygote was detected by Sanger sequencing (Reference cDNA sequence: NM_000135.2) (Figure 1). The above sequence variant was identified in heterozygous state in his parents. Further, the above sequence variant was not identified in other family members (elder sibling, maternal aunt and cousin).

The variant found in our study is submitted to ClinVar. It is a freely accessible, public archive of reports of the relationships among human variations and phenotypes hosted by the National Center for Biotechnology Information (NCBI) and funded by intramural National Institutes of Health (NIH) funding. The variant submission accession number is SCV000189618 (http://www.ncbi.nlm.nih.gov/clinvar/variation/156400/).

## Discussion

Fanconi anemia is a rare hereditary disorder and characterized by bone marrow failure, chromosome breakage and development of cancer. We report a case of FA with classical presentation. The genetic investigation is a rapid method to identify deletions and duplications, enabling faster identification of mutations in FA. A large spectrum of mutations has been reported in the *FANCA* gene, including microdeletions, large deletions, microinsertions and point mutations [8-10,15].

In our case, molecular genetic testing by mutation analysis in 15 *FANC* genes, exhibited a novel and homozygous splice site mutation, c.3066 + 1G > T, (IVS31 + 1G > T) in *FANCA* and parents were found to be heterozygous for the same mutation, confirming the diagnosis of FA. However, since defects in the *FANCA* gene are reported in high frequency of FA patients and large deletions are the most common mutations in the gene, molecular genetic testing by mutation analysis has been found to be a very useful technique for the rapid detection of genetic changes in FA. Identification of the mutation expands the mutational spectrum of FA, facilitates prenatal diagnosis and helps in taking decisions on treatment and management of the disease. This is the first time in the world that this novel mutation is reported in Fanconi Anemia. The variant submission accession number is SCV000189618 (http://www.ncbi.nlm.nih.gov/clinvar/variation/156400/).

### Review of literature

FA was first described in 1927 by the Swiss Paediatrician Guido Fanconi. It is a rare genetically inherited autosomal disorder characterized by congenital malformations, progressive pancytopenia, cellular hypersensitivity to DNA-cross-linking agents, predisposition to acute myelogenous leukemia (AML) and other malignancies [1]. The developmental and physical abnormalities may include hyperpigmentation, short stature, malformations of the thumb and forearms, skeletal anomalies, small head or eyes, renal problems, hearing defect, heart disease, gastrointestinal difficulties and hypogonadism. The incidence of FA is approximately 1 to 5 per million [16]. This condition is more common among the people of Ashkenazi Jews, with carrier frequency of 1 in 89 [17], and black South Africans where the carrier frequency is 1 in 83 [18].

The genes that have found to be mutated in FA patients are called *FANC*. The genes *FANCA, FANCB, FANCC, FANCD1 (also known as BRCA2), FANCD2, FANCE, FANCF, FANCG, FANCI, FANCJ, FANCL, FANCM, FANCN, FANCO, FANCP, and FANCQ* have been identified as responsible for FA [2-5]. All the *FANC* genes are autosomal recessive except *FANCB*, as this gene is located on the X chromosome [19]. Mutations in *FANCA*, *FANCC, FANCG and FANCD2* are the most frequent among FA patients worldwide [20]. Among these *FANCA* gene abnormalities account for approximately 60–70% of FA patients and to date more than 100 types of mutations have been found throughout the *FANCA* gene [6-9]. Most of these mutations may lead to premature termination or intragenic large deletions, presumably lack protein expression (null-mutations) and altered proteins with a single amino acid substitution [8-10].

### *FANCA* gene

The *FANCA* gene is located at 16q24.3; more precisely it is located from base pair 89, 737, 550 to base pair 89, 816, 657 on chromosome 16 and contains 43 exons spanning 34 to 188 base pairs (Reference sequence NM_000135.2). It is a relatively large protein consists of 1455 amino acids (163 kDa) [5,21].

**Figure 2:**
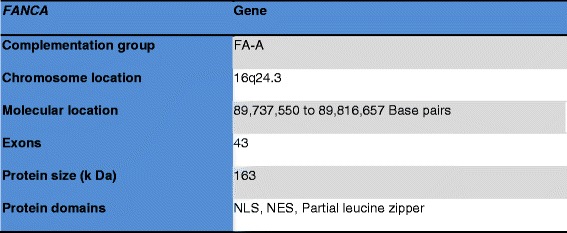
*FANCA* gene details.

*FANCA* contains a nuclear localization signal (NLS) at its aminoterminus, a partial leucine zipper like motif between aminoacids 1069 and 1090 and a five functional leucine-rich nuclear export sequences (NESs) [6,22,23] (Figure 2).

The other names of *FANCA* are *FA, FA1, FAA, FACA, FANCA_HUMAN, FA_H, FAH, and FANCH. FANCA* is implicated in the complementation group A (FA-A), the most frequent complementation group accounting for about 70% of FA cases. Sequencing the *FANCA* gene of FA-A patients has demonstrated numerous *FANCA* mutations and polymorphisms. Significant diversity of *FANCA* mutation in the type and location have been detected, including insertions, deletions, nonsense, mis-sense, splicing, frameshift mutations [16-20]. As the large number of *FANCA* mutations and polymorphism have been identified, the *FANCA* is considered as hypermutable and highly polymorphic gene [2,6,7,9-12].

### *FANCA* protein

*FANCA* is a multi-subunit complex composed of *FANCA, FANCC, FANCF* and *FANCG* proteins. Fanconi Anemia genes are functionally related. *FANCA*, *FANCC* and *FANCG* proteins are components of a nuclear protein complex. *FANCA* and *FANCC* bind each other and form a *FANCA*-*FANCC* complex. *FANCG* is required for binding of *FANCA*-*FANCC* complex. This complex is found in abundance in both cytoplasm and nucleus [24]. In turn *FANCA* amino terminal nuclear localization signal is required for *FANCG* binding and *FANCC* binding. The *FANCA* and *FANCG* interaction *is* constitutive and is not regulated by *FANCC* or by the products of other FA genes. Whereas, the binding of *FANCC* requires *FANCA/FANCG* binding and the products of other Fanconi Anemia genes [25].

**Figure 3:**
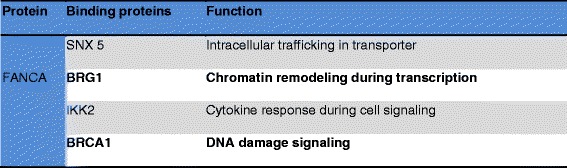
FANCA- binding proteins.

*FANCA* interacts with some of the non FA protein such as Sorting Nexing protein (SNX5), Brahma-Related Gene 1 protein (BRG1), IκB Kinase-2 (IKK2), Breast Cancer 1 (BRCA1) and involves in various functions (Figure 3).

SNX5 is a *FANCA*-binding protein. The interaction of *FANCA* with the sorting nexin 5 protein (SNX5) may be involved in its subcellular trafficking. *FANCA* protein levels increases during overexpression of SNX5 and traffic with cell surface receptors could be affected by *FANCA* [26].

*FANCA* interacts with BRG1, a major coregulator of transcription, both in activation and repression, through chromatin modulation. BRG1 is a subunit of the SWI/SNF complex; *FANCA* may take on, this complex to target genes to enable transcription and DNA repair mechanism [27]. *FANCA* transcripts are seen in the brain, kidney, liver whisker follicles, retina and teeth. In *FANCA* expression there is a stage-specific variation, predominantly within the developing whiskers and the brain. Some tissues express *FANCC,* but they are unable to show *FANCA* expression. Thus, *FANCA* is not necessarily coupled to that of *FANCC* [28].

*FANCA* was also found to associate with the IKK signalosome through direct interaction with the IKK2 [29]. It remains unclear that, though the phosphorylation state of several *FANCA*-associated proteins has been affected by the kinase activity, the direct phosphorylation of *FANCA* by IKK2 has been reported. Additionally, FANCA binds to the tumor suppressor BRCA1 and involves in DNA damage signalling.

### *FANCA* functions

The *FANCA* gene encodes a protein that is involved in a cell process known as the Fanconi anemia (FA) pathway. The FA pathway has a specific role in certain type of DNA damage known as interstrand cross-links (ICLs) [30-32]. The *FANCA* protein is a component of FA core complex. FA core complex activates *FANCD2* and *FANCI* proteins by a process called monoubiquitination and forms the *FANCD2-FANCI* (ID) protein complex, this complex co-localizes the DNA repair proteins to the DNA damage site as a result the error can be rectified and DNA replication can continue [33,34].

*FANCA* phosphorylation is necessary for the formation of FA complex and nuclear localization. Despite the fact that *FANCA* phosphorylation is required for normal *FANCA* function, the specific *FANCA* phosphorylation site(s) and the kinase(s) responsible for *FANCA* phosphorylation are unknown [35,36].

The *FANCA-FANCG* interaction is required for genomic stability. *FANCA* protects humans from bone marrow failure and malignancies by maintaining genomic integrity. *FANCA* is primarily localized in the nucleus. It interacts with cytoplasmic proteins as well as nuclear proteins, but some data suggest that *FANCA* has a distinct cytoplasmic function in addition to a nuclear function [29,37,38].

To date, little has been learned regarding the regulation of *FANCA* expression, protein-protein interactions, and nuclear localization. Multiple *FANCA* transcripts have been recognized by Northern blotting technique, suggesting that *FANCA* gene may be involved in the regulation of *FANCA* expression through the mechanism of alternative splicing [21].

### *FANCA* mutations

*FANCA* is the largest and the most polymorphic FA gene. FA-A is the most prevalent complementation group therefore mutation analysis of FA patients with undefined complementation group is always started in *FANCA* gene. Mutation analysis can be performed on genomic DNA as well as on cDNA [7].

The mutation spectrum in the *FANCA* gene is very heterogenous and also it varies from *FANCC* and *FANCG*. More than 100 different mutations that have been dispersed in the exons and introns of the entire *FANCA* gene of the FA patient are described in some studies [7,8,21]. To the best of our knowledge there are very few studies with the mutation detection in FA.

The hyper-mutability of *FANCA* is due to the dispersion of large number of repetitive elements throughout the gene. Repetitive elements include *Alu*-repeats, short direct repeats, CpG-islands homonucleotide tracts and several hotspot-motifs such as CCTG/CAGG. The *FANCA* gene possesses all kinds of mutations. The most frequent mutation includes large deletions which may span 1-31 exons. In some reports even the deletions spanning entire *FANCA* genes have also been described [39]. Some of the Splice site mutations, nonsense and missense mutations have also been reported. Since the Patients carrying same mutations and homozygosity are found rarely, most of the *FANCA* mutations are called “private mutations”, except few common mutations [15].

### Fanconi anemia reports from India

In a report from India in 2014, molecular analysis of proband (A 5-year-old girl having hypoplastic anemia) showed heterotzygous c.1303C > T (rs148473140: R/C) mutation in FANCA gene. Father was found to be carrier for the mutation and the mother was normal [38].

In a conference abstract out of a Indian study by Arthur et al., 2014 [40] a comprehensive clinical and molecular analysis in 101 patients with pancytopenia was performed. A total of 76 patients had characteristic physical abnormalities of FA, of which perioral hyperpigmentation (42%) was the most common in these patients. The median age at diagnosis was 11 years (range 4-30) and sex ratio between males to females was 3:2. The chromosome breakage analysis (CBA) was performed on peripheral blood samples at diagnosis. CBA score greater than the cut off value of 40 was seen in 63% (n = 48/76) of the patients. FANCD2 ubiquitination investigation of these patients with physical abnormalities in peripheral blood and fibroblasts showed absence of ubiquitination in 69 (90%) of the patients while the control group had normal ubiquitination pattern. In order to further characterize the FA subtypes, fibroblasts from 20 patients were transduced with lentiviral *FANCA* and in 17 (85%) FANCD2 ubiquitination could be restored suggesting a high frequency of *FANCA* defects in Indian population. Mutation screening of *FANCA* in a subset of patients showed large deletions in five of which three are novel (ex 10_37 del,ex 6_14 del and ex 1_4 del). There was a deletion of two nucleotides resulting in a frameshift mutation in one patient (c.3760_3761delGA) and a missense mutation (c.2786A > C) in another. Our data suggests that FANCD2 ubiquitination analysis in conjunction with CBA is useful for the diagnosis of FA and detection of mosaicism and the complementation analysis shows high frequency of *FANCA* defects in patients with FA in Indian population [40].

In a proceeding of a conference, Vundini in 2014, [41] reported molecular study of FA in the Indian population. FA was screened by CBA using MMC and diepoxybutane (DEB) induction and FANCD2 monoubiquitination by western blotting to understand gene defects in FA pathway. The complementation analysis was done by retroviral transfection. The molecular study was carried out by Multiplex Ligation-dependent Probe Amplification (MLPA) and direct sequencing of *FANCA*, *C*, *G*, *E*, *F*, *L*, and *M* genes. The study showed FA-A and E gene defects in 69% cases and followed by *FANCE* gene defect. The molecular analysis showed the large deletions in 11 patients and *FANCA* gene mutations were in 12 FA patients. Out of 12 mutations 5 mutations (c.3678 C > G, p.Ser 1226 X, c.3993G > A p.Leu 1331 Pro, c.1274 C > G p.Glu 425 His, c.2630 C > G p.Ser 877 X) found to be novel mutations. In the series the single nucleotide polymorphisms (SNP); Exon9,c.796A > G(p.Thr266Ala), Exon16,c.1501G > A(p. Gly501Ser), Exon26, c.2426G > A(p.Gly809Asp), of *FANCA* gene also observed in 23 patients, and these polymorphisms in disease association was reported in FA database. Interesting finding of this study is the existence of *FANCE* mutation in Indian population, and the same was reported rarely in other places of the world. The study also highlighted the uncharacterized FA patients, which may be associated with new genes in Indian population and these patients should be studied molecularly and genotype-phenotype correlation need to be established, which assist in better understanding and supervision of the disease [41].

In a retrospective study carried out in New Delhi, 528 aplastic anemia patient samples were tested for CBA. A significant increase in chromosomal breakages was seen in 13.1% patients. The survival data documented for 100 patients suggested 60% mortality [42].

In a case report of FA, the CBA together with FANCD2 Western blot monoubiquitination assay confirmed the diagnosis as FA. MLPA revealed a novel homozygous large intragenic deletion (exons 8-27 del) in the *FANCA* gene in the subject. His siblings and parents were also analyzed and found to be heterozygous for the same mutation [43].

In a report in 2010, CBA using DEB induction was performed in 195 pediatric patients suspected with FA. CBA showed 33 (17%) patients with classical FA, 9 (4%) with somatic mosaicism FA, 25 (13%) with FA with high frequency of chromosomal breakage and without clinical features, and 128 (66%) with suspected FA but had no chromosomal breakage and clinical features of FA. CBA is an important investigation tool for differentiating FA from idiopathic aplastic anemia [44].

Korgaonkar et al. performed study in 33 clinically diagnosed FA patients having aplastic anemia and bleeding abnormalities. The genetic analysis revealed a significantly (P < 0.0001) high frequency (36.4%) of parental consanguinity in FA patients. CBA revealed spontaneous chromosome breakage in 63.64% FA patients. Among 33 patients, nine (27.27%) patients developed malignancies and chromosomal abnormalities were detected in five (55.5%) patients bone marrow cells including monosomy 5 and 7, trisomy 10, der(1q) and inv(7). Cytogenetic investigation is important in aplastic anemia to rule out FA [45].

Varma et al. observed 24.07% (13/54) of FA among bone marrow failure syndrome (BMFS) patients. Authors found DEB induction a better test than MMC test in screening FA [46].

In a report of FA in newborn presented with anophthalmia, unilateral radial ray defect, hemivertebrae and thrombocytopenia, CBA revealed a high frequency of chromosomal breakage (6.0/metaphase). Chromosomal analysis of siblings and parents revealed normal karyotype. In view of poor long term prognosis, parents refused any further treatment and baby was discharged against medical advice [47].

## Conclusion

It is concluded that genetic study should be done if possible in all the cases of suspected FA, siblings, parents and close blood relatives. The screening of the *FANCA* gene for mutations supports the clinical diagnosis of FA. Further, it will help us to plan appropriate treatment and also to select suitable donor for hematopoietic stem cell transplantation and to plan for genetic counseling. Future studies would clearly advance our understanding of FANCA regulation and function.

## Consent

Written informed consent was obtained from the patient’s guardian/parent/next of kin for the publication of this report.
